# Bioanalytical UHPLC–MS/MS Method for Quantification of Terbinafine in Ungual Delivery Studies

**DOI:** 10.1002/bmc.70392

**Published:** 2026-02-12

**Authors:** Benjamin Rossier, Emmanuel Varesio, Eric Allémann, Yogeshvar N. Kalia

**Affiliations:** ^1^ School of Pharmaceutical Sciences University of Geneva Geneva Switzerland; ^2^ Institute of Pharmaceutical Sciences of Western Switzerland Geneva Switzerland

**Keywords:** fractional laser ablation, nail, onychomycosis, terbinafine, UHPLC–MS/MS, ungual drug delivery

## Abstract

Terbinafine is considered the first‐line treatment for dermatophyte‐induced onychomycosis. This study aimed to develop a fast, selective, and sensitive UHPLC–MS/MS method for the quantification of terbinafine in porcine hooves, a cost‐effective surrogate for human nails. The objectives were to (i) optimise specific chromatographic and detection settings, (ii) evaluate terbinafine extraction recovery, (iii) validate the method with regard to standardised operating procedure guidelines using terbinafine‐d3 as an internal standard and (iv) investigate the method's performance through a preliminary study involving deposition of an in‐house high‐loading topical formulation into fractional laser‐ablated porcine hooves. An extraction with MeOH:H_2_O (9:1) achieved ~90% recovery. Extracts were centrifuged, filtered and analysed under gradient conditions, with analyte and internal standard detected by selected reaction monitoring. The validated method demonstrated appropriate sensitivity to quantify concentration ranges between 1 and 200 ng/mL with a 7.2‐min runtime and a limit of quantification of 1 ng/mL. Preliminary studies detected terbinafine from the formulation within intact and laser‐ablated porcine hooves, corresponding to deposited amounts of 89.04 ± 4.02 and 217.49 ± 8.98 μg/cm^2^, respectively. In conclusion, the sensitivity and specificity of the method make it suitable for use in further investigations into ungual delivery enhancement strategies involving terbinafine formulations.

## 1 Introduction

**FIGURE 1:**
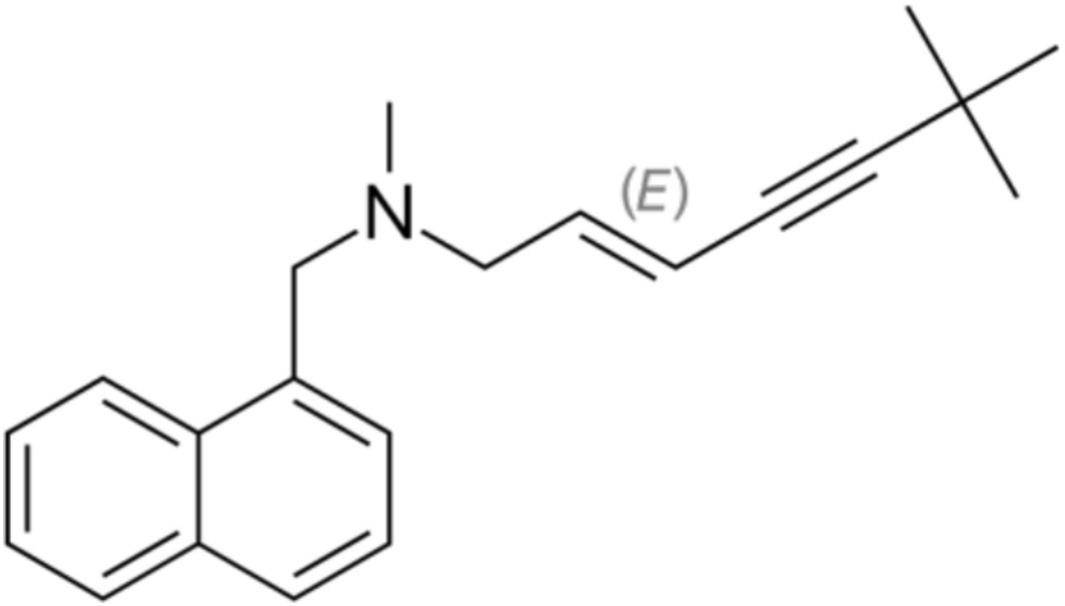
Chemical structure of terbinafine.

Terbinafine ((*E*)‐*N*‐(6,6‐dimethyl‐2‐hepten‐4‐ynyl)‐*N*‐methyl‐1‐naphthalene methanamine) (TBF) is a synthetic allylamine derivative (Figure 1) commonly used to treat cutaneous and ungual fungal infections due to dermatophytes, the predominant pathogens responsible for onychomycosis (Gupta et al. 2003). Its fungicidal action relies on blocking ergosterol synthesis through inhibition of squalene epoxidase, thus preventing synthesis of the fungal cell wall (Gokhale and Kulkarni 2000). Although TBF can be administered orally or topically, in principle, there is a clear preference for the latter given the localised nature of the disease and accessibility to the site. However, the structural complexity and limited permeability of the nail plate (S. Baswan et al. 2017; Gupta et al. 2023; Kobayashi et al. 2004) limit the penetration of TBF from topical formulations, underlining the need for a sensitive, robust and reproducible analytical method for its quantification in the nail matrix.

Given the issues related to the availability of human nails, and the ethical limitations and potential risks associated with using infected human nails, porcine hooves have emerged as a robust surrogate for studying ungual drug delivery in preclinical studies. Despite differences in morphology and composition (S. M. Baswan et al. 2016; Thatai and Sapra 2014), their structural similarity to human nails and their accessibility provide an ethical and practical alternative for assessing the efficacy and penetration of topical antifungal agents (Eurell 2004). Furthermore, the increased thickness and porosity observed in onychomycotic toenails make the comparison with this model more suitable than healthy human nails.

Several groups have developed chromatographic methods to quantify TBF in biological tissues, including high‐performance liquid chromatography (HPLC) coupled with a UV detector, diode array detector (DAD) or gas chromatography (GC) with a flame ionisation detector (FID) (Baranowska et al. 2009, 2010; Denouël et al. 1995; Dykes et al. 1990; Miron et al. 2014; Ünal 2010; Zehender et al. 1995). Although these techniques are suitable for routine quantification, achieving precise measurements can be challenging when dealing with low‐concentration samples or using complex matrices. For several decades, liquid chromatography hyphenated to mass spectrometry (LC–MS) has proven effective for detecting analytes in complex samples, thanks to its high throughput and robustness (Hopfgartner and Bourgogne 2003). For quantitative analyses, multiple reaction monitoring (MRM) performed on triple quadrupole systems remains the method of choice due to its sensitivity and selectivity. More recently, QUAL/QUAN workflows have emerged as an alternative to LC–MRM techniques, taking advantage of the high resolving power of the new hybrid mass spectrometers like Qq‐TOF or orbitraps (Hopfgartner et al. 2012). However, in LC–MS the quantification performance is sometimes impaired by electrospray ionisation suppression due to a matrix effect (ME), thus requiring an efficient preliminary sample preparation or a multidimensional separation to improve selectivity (Abouir et al. 2024; Sparkman et al. 2011).

Several LC–MS studies have reported the quantification of TBF in various biological matrices, including human plasma, minipig plasma and human skin and hair (Brignol et al. 2000; de Oliveira et al. 2001; Dotsikas et al. 2007; Gou et al. 2022; Majumdar et al. 2000). Concerning nails, studies were mostly performed using HPLC–UV (Denouël et al. 1995; Dykes et al. 1990; Zehender et al. 1995). More recently, a study by Davies‐Strickleton et al. (2020) investigated the nail penetration of various antifungal drugs, including TBF, using healthy human nail clippings mounted in Franz cells. For this study, they developed and validated a single UHPLC–MS/MS method to selectively quantify the ungual deposition and transungual permeation of the different drugs. Although their protocol enabled the separation and simultaneous quantification of five molecules in nail lysates and the receiver compartment, the method was not specifically optimised for TBF. The data demonstrated the reduced stability of the compounds following sample preparation using 5‐M NaOH, with a recovery efficiency of less than 40% for TBF. In addition, the extraction procedure involved dissolution of the nail before quantification without filtration, increasing the amounts of endogenous compounds in the samples and raising the risk of unwanted interaction with the analyte. Therefore, it would be of significant interest to develop a procedure with a higher extraction efficiency for TBF and which avoids the use of harsh conditions.

The aim of this study was to develop and validate a fast and sensitive UHPLC–MS/MS analytical method for the quantification of TBF in porcine hooves. To minimise the influence of endogenous compounds, an alternative extraction method was also developed instead of using nail lysate. In order to verify the applicability of the method presented here, a preliminary study was conducted to quantify the deposited amount of TBF in laser‐ablated porcine hooves following the application of a TBF nanocrystal‐loaded thermosensitive gel. The long‐term objective is to use this validated methodology to extract and quantify TBF in future projects dealing with infected human toenails.

## 2 Experimental

### 2.1 Chemicals and Reagents

TBFHCl was purchased from Sigma‐Aldrich (Saint‐Louis, MO, USA). TBF‐d3–HCl was purchased from Cayman Chemical Company (Ann Arbor, MI, USA) and used as internal standard (IS). Ultrapure water (H_2_O) was obtained from a Milli‐Q water purification system (resistivity > 18 MΩ.cm) from Merck Millipore (Darmstadt, Germany). Methanol (MeOH, LC–MS grade) and acetonitrile (ACN, LC–MS grade) were obtained from Fisher Scientific (Waltham, MA, USA). Formic acid (FA, UPLC/MS grade) was purchased from Biosolve (Dieuze, France). TBF commercial formulation (Terbinafine‐Mepha Crème 10 mg/1 g, Mepha, Aesch, Switzerland) was purchased from a local pharmacy. Poloxamer 407 (P407) was obtained from Sigma‐Aldrich (St. Louis, MO, USA).

### 2.2 Liquid Chromatography

The liquid chromatographic system was composed of an Acquity UPLC (Waters, Baden‐Dättwil, Switzerland), including a binary solvent manager, a column manager and a sample manager with an injection loop volume of 10 μL. The reversed‐phase chromatographic quantification of TBF was carried out using a XBridge BEH C_18_ column (100 × 2.1 mm, 2.5 μm) equipped with its 5‐mm VanGuard precolumn (Waters). A gradient elution was performed using mobile phases consisting of 0.1% FA in water (A) and 0.1% FA in ACN (B). Separation was carried out at a flow rate of 0.4 mL/min using a linear gradient 20%–100% of Mobile Phase B in 4 min followed by a wash step of 2 min before column reconditioning (overall runtime of 7.2 min). The column temperature was set to 40°C, and the sample manager was kept at room temperature. Sample injection volume was 5 μL (partial loop injection mode).

### 2.3 Mass Spectrometry

The UHPLC system was hyphenated to a Xevo TQ‐S micro triple quadrupole mass spectrometer (Waters, Baden‐Dättwil, Switzerland). MS detection was carried out using electrospray ionisation in positive polarity (ESI+) and selected reaction monitoring (SRM). Electrospray voltage was set to 3.50 kV; cone voltage at 20 V; desolvation gas (nitrogen) and cone temperature were 500°C and 150°C, respectively; and the gas flow was maintained at 1000 L/h. SRM transition and specific MS parameters of each compound were tuned and determined by infusing the analyte and the IS individually (1 μg/mL in MeOH) at a flow rate of 5 μL/min. Collision energy was adjusted to optimise signal intensity of each compound, and transition dwell time was set to 61 ms to obtain ~15 data points across the LC peak width (at base). Data acquisition and processing were performed using MassLynx software version 4.2 (Waters).

### 2.4 Preparation of Porcine Hoof Matrix

Fresh porcine hooves were obtained from a local slaughterhouse (Abattoir de Loëx Sarl, Bernex, Switzerland). The hooves were softened by soaking them for 5 min in water; then, the lateral part was removed and cut to obtain square‐shaped pieces of approximately 16‐mm length. Each piece was cut into smaller pieces in a flask containing 10‐mL MeOH:H_2_O (9:1) and left for extraction overnight under agitation at room temperature. After 24 h, the extraction mixture was transferred into tubes prior to centrifugation at 12,000 *g* for 20 min using a Sorvall Lynx 4000 centrifuge (ThermoFisher Scientific, Waltham, MA, USA) and filtered through 0.2‐μm Chromafil Xtra polytetrafluoroethylene (PTFE) membrane filters (Macherey‐Nagel GmbH, Düren, Germany). The supernatants were collected and stored at −20°C.

The porcine hoof matrix solution was used to assess both the method's specificity and the ME. A set of calibration standards and quality control (QC) samples was prepared in the porcine hoof matrix as well.

### 2.5 Preparation of Stock Solutions, Calibration Standards and QC Samples

Individual stock solutions were prepared at 1 mg/mL in ACN:H_2_O (1:1) for TBF and IS. The analyte and IS stock solutions were freshly prepared before each analysis. Two working solutions of analyte were prepared by dilution in ACN:H_2_O (1:1) for one set of calibration standards and in the hoof matrix solution for the other set. Both working solutions were diluted at a concentration of 1 μg/mL. Two working solutions of the IS were also prepared at 1 μg/mL in ACN:H_2_O (1:1) and the hoof matrix solution.

Two different sets of calibration standard samples were prepared by dilution of the two working solutions in their respective solvents in a concentration range from 1 to 200 ng/mL. An appropriate volume of the working solution of IS was used to spike the calibration standard samples to obtain a final concentration of IS of 10 ng/mL.

QC samples were prepared using the same method at 25, 75 and 150 ng/mL.

### 2.6 Method Validation

The analytical method and extraction procedure of the analyte from the porcine hoof were assessed based on guidelines such as the ICH Q2(R2) guidelines (2023 version). However, full validation according to the ICH M10 guidelines would be required to support regulatory submissions.

#### 2.6.1 Selectivity, Specificity and Carryover

The selectivity of the method was ensured by using specific SRM transitions to quantify the analyte and IS. For this, SRM transitions of both compounds were compared at 10 ng/mL in ACN:H_2_O (1:1). Then, specificity was confirmed with regard to the endogenous compounds present in the porcine hoof, ensuring no interference between their peaks and those of the analytes. For this purpose, a blank porcine hoof matrix sample was analysed and compared with a porcine hoof matrix spiked with the analyte at 1 ng/mL.

The carryover was verified for each experiment by injecting a blank hoof matrix after the highest concentration standard (i.e., 200 ng/mL).

#### 2.6.2 ME Evaluation

Signal enhancement/suppression related to the ME was investigated by comparing the signal obtained with the QC prepared in a solution of ACN:H_2_O (1:1) versus the signal of that prepared in the porcine hoof matrix. Each sample contained IS at 10 ng/mL. According to the definition proposed by Matuszewski et al. (2003), ME was evaluated using the following equations:

(1) $$\mathrm{ME}\;\left(\%\right)=\frac{\text{Analyte area spiked matrix}}{\text{Analyte area standard solution}}\times 100\%$$

(2) $$\%\text{IS}‐\text{normalised matrix effect}=\frac{\text{Matrix factor anal}y\mathrm{te}}{\text{Matrix factor IS}}\times 100\%$$

#### 2.6.3 Extraction Procedure Efficiency

The efficiency of the extraction procedure to recover the analyte from the porcine hoof was evaluated and validated using porcine hoof samples obtained with the procedure described in Section 2.4. Porcine hoof pieces (*n* = 3 per concentration) were spiked with 10 μL of a TBF methanolic solution at 1, 10 and 100 μg/mL, then left for evaporation. Afterwards, samples were cut into small pieces, placed in flasks containing 10‐mL MeOH:H_2_O (9:1) and extracted overnight under agitation at room temperature. Extracts were centrifuged at 12,000 *g* for 20 min and filtered to collect the supernatant for LC–SRM analysis. Each concentration was diluted in porcine hoof matrix solution into vials to reach a theoretical final concentration of 100 ng/mL (corresponding to 100% recovery). Each sample was spiked with 10 ng/mL of IS.

The extraction efficiency was determined by calculating the recovered amount to the spiked amount as shown in the following equation:

(3) $$\text{Extraction efficiency}\;\left(\%\right)=\frac{\text{Recovered amount}\times \text{dilution factor}}{\text{Spiked amount}\;}\times 100\%$$

#### 2.6.4 Calibration Curve Range and Response Linearity

Calibration standards were prepared by spiking the analyte at known concentrations in a standard solution of ACN:H_2_O (1:1) and in porcine hoof matrix obtained as described in Section 2.4. The concentrations were selected to cover a detectable range of concentrations and avoid saturation. Eight calibration standards containing 10 ng/mL of IS were prepared at 1, 2, 5, 10, 20, 50, 100 and 200 ng/mL (*n* = 3) in solutions mentioned above as described in Section 2.5.

The calibration curves were plotted with the response as a function of the analyte concentration. The response for each sample was automatically calculated using the following equation:

(4) $$\text{Response}=\text{Area}\times \frac{\text{IS concentration}}{\text{IS area}}$$

The acceptance requirement for the linearity of a calibration curve was a correlation coefficient (*R* ^2^) of at least 0.99 and an RSD < 20% at the lower limit of quantification (LLOQ).

#### 2.6.5 Limit of Detection (LOD) and Limit of Quantification

The LLOQ was determined in two steps. First, the difference between the predicted area of the analyte, ranging from 1 to 200 ng/mL with 10‐ng/mL IS, and the experimental area was calculated using the following equation:

(5) $$\frac{\text{Area analyte}}{\text{Area IS}}=\text{slope}\times \frac{\left[\text{Analyte}\right]}{\left[\text{IS}\right]}+\text{Intercept}$$

Then, the average standard deviation of the response (*σ*) was calculated by the difference between the experimental area and the predicted area for all concentrations. Eventually, the LLOQ was determined using the following equation:

(6) $$\text{LLOQ}=10\times \frac{\sigma }{\text{slope}}$$

The LOD was determined by following the same procedure and was calculated as follows:

(7) $$\mathrm{LOD}=3.3\times \frac{\sigma }{\text{slope}}$$

#### 2.6.6 Accuracy and Precision

Accuracy, expressed as percentage recovery, and precision of three QCs containing 25, 75 and 150 ng/mL of analyte (*n* = 3) were assessed over 3 days.

Accuracy was calculated as the ratio of the measured concentration, calculated using the calibration curve obtained on the same day of the analysis, with the actual spiked concentration following the equation below:

(8) $$\text{Accuracy}=\frac{\text{Measured concentration}}{\text{Actual concentration}}\times 100\%$$

Intra‐day accuracy was evaluated by calculating the percentage recovery of the same concentration measured from three samples during the same day. Interday accuracy was evaluated by calculating the percentage recovery of three independent samples at the same concentration over 3 days.

Intra‐day precision was evaluated by calculating the relative standard deviation (RSD) of the same concentration measured from three samples during the same day. Interday precision was evaluated by calculating RSD of three independent samples at the same concentration over 3 days.

### 2.7 Preliminary Study—Application of the Analytical Method

The applicability of the method was assessed through a preliminary study using single application of a high‐loading 10% (w/w) TBF nanocrystal‐loaded P407 gel formulation prepared in‐house (TBF NC gel) and a conventional TBF commercial formulation 1% (w/w) (Terbinafine‐Mepha Crème 10 mg/1 g) on laser‐ablated and intact porcine hooves. Briefly, porcine hoof pieces (*n* = 3) were prepared as outlined in Section 2.4. The dorsal surface was microporated using a Ultrapulse CO_2_ fractional laser system (Lumenis Ltd., Santa Clara, CA, USA) equipped with Synergistic Coagulation and Ablation for Advanced Resurfacing (SCAAR) FX and Deep FX modes. SCAAR FX mode was used with parameter settings of 80‐mJ energy, 250 Hz, 10% fractional ablation area (number of pores per unit surface area) and one pulse per pore. Then, 10 μL of formulation was applied on the dorsal surface, gently rubbed to facilitate penetration into the micropores, and the formulation excess was removed with a cotton swab. Samples were prepared following the protocol detailed in Section 2.4 and diluted within the validated concentration range based on the approximate TBF concentration applied. To prevent interference, the emulsion of the commercial formulation was disrupted by heating at 60°C and sonicated for 10 min after soaking in the extraction mixture. Results obtained with laser‐ablated porcine hooves were compared with those from intact samples, which served as controls.

### 2.8 Statistical Analysis

Data are expressed as means ± SD. Statistical differences were determined by two‐way ANOVA (intact vs. laser‐ablated hooves and TBF NC gel vs. commercial cream), followed by Šídák's multiple comparison test, which was performed using GraphPad Prism software 10.2.3 (GraphPad Software, Boston, MA, USA).

## 3 Results and Discussion

### 3.1 Method Development

Chromatographic conditions were refined by optimising the gradient to perform the analysis within the shortest possible runtime, including a 2‐min washing step to minimise carryover. In parallel, SRM transitions were optimised to provide optimal sensitivity and LC peak shape.

Retention time of the analyte and IS was 2.95 min with a flow rate of 0.4 mL/min, a column temperature of 40°C and 5‐μL injection volume. These chromatographic conditions were selected and used for the subsequent validation of the analytical method.

TBF is a weak base, with a calculated pKa of 7.5 for its tertiary amine moiety. Therefore, electrospray was operated in positive polarity to ionise TBF and TBF‐d3 as their protonated adduct ([M + H]^+^). SRM detection was selected for its sensitivity and specificity to reduce the risks of interference.

The precursor ions corresponding to TBF and TBF‐d3 were observed at *m/z* 292.1 and 295.2, respectively. Two fragment ions were selected at *m/z* 114.9 and 141.0 for both compounds to obtain better selectivity, but only the transitions involving the fragment at *m/z* 141.0 were used for the quantification. Table 1 summarises the optimised chromatographic conditions and SRM settings.

**TABLE 1:** Chromatographic conditions and SRM settings for TBF and TBF‐d3.

LC parameters	
Column	C_18_ 2.1 × 100 mm × 2.5 um
Precolumn	C_18_ 2.1 × 5 mm × 2.5 um
Mobile phases	Water/acetonitrile 0.1% FA
Column temperature (°C)	40
Flow rate (mL/min)	0.4
Volume of injection (μL)	5
Sample temperature (°C)	25
Run time (min)	7.2
Mode	Gradient

MS/MS parameters	Terbinafine	Terbinafine‐d3
Nature of parent ion	[M + H]^+^	[M + H]^+^
Precursor ion (*m/z*)	292.1	295.2
Product Ion 1 (*m/z*)	141.0	141.0
Product Ion 2 (*m/z*)	114.9	114.9
Collision energy (V)	20	
Cone voltage (V)	22	
Electrospray mode	Positive	
Electrospray voltage (kV)	3.5	
Source temperature (°C)	150	
Desolvation temperature (°C)	500	
Desolvation gas flow (L/h)	1000	
Collision gas flow (L/h)	1	

### 3.2 Method Validation

#### 3.2.1 Specificity/Selectivity

**FIGURE 2:**
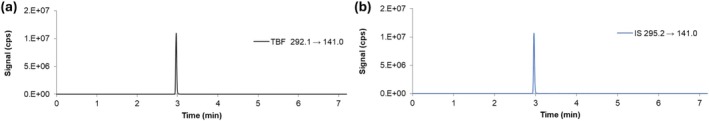
LC–SRM traces of TBF (a, 292.1 > 141.0) and IS (b, 295.2 > 141.0, TBF‐d3) standard solution (10 ng/mL in ACN:H_2_O [1:1]).

No interference was observed after a single injection of the standard solution of the analyte and the IS in ACN:H_2_O (1:1) (Figure 2). Comparison of the two chromatograms showed that the retention times matched with good peak resolution. The contribution of the IS to the TBF signal was estimated to be 0.39% (Supporting Information).

**FIGURE 3:**
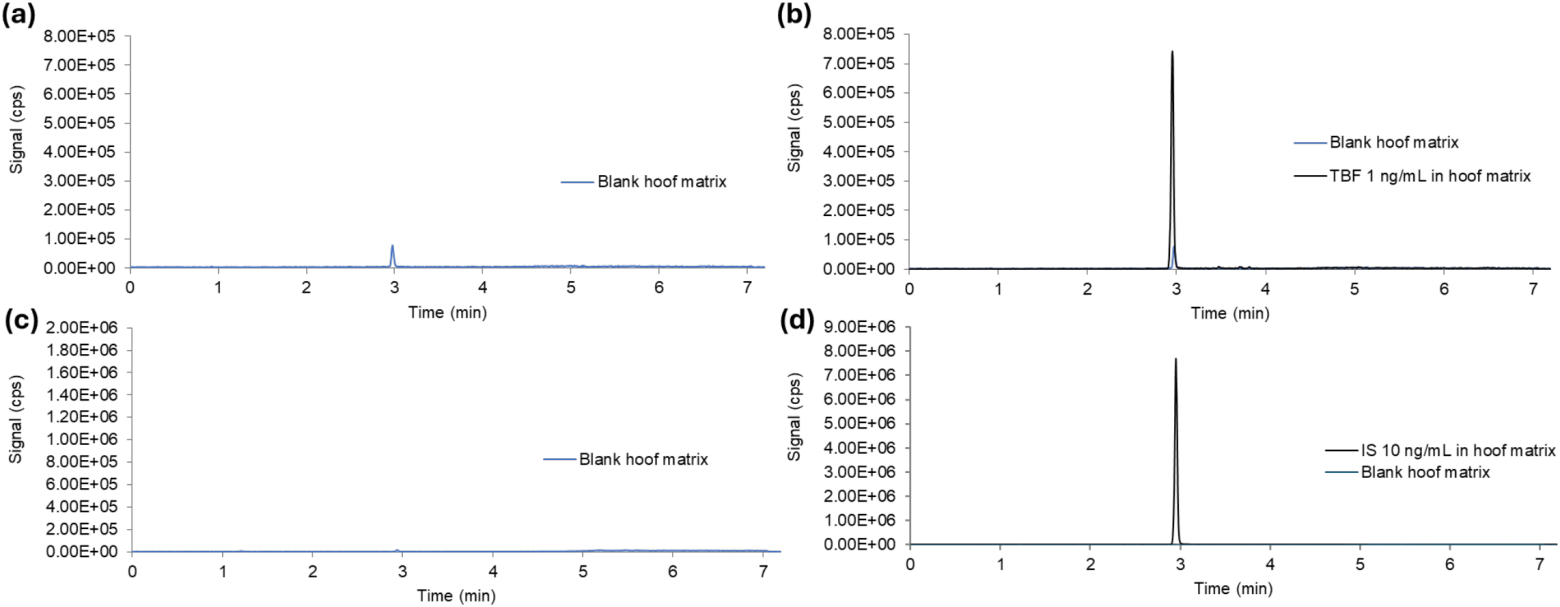
LC–SRM traces (TBF, 292.1 > 141.0) of (a) blank and (b) TBF‐spiked porcine hoof matrix and porcine hoof matrix (1 ng/mL). LC–SRM traces (TBF‐d3, 295.2 > 141.0) of (c) blank and (d) IS‐spiked porcine hoof matrix (10 ng/mL).

Comparison between the blank hoof matrix sample and the sample spiked with the analyte or the IS showed no interference from the endogenous components of the hoof matrix and the analytes because the retention time did not change (RT = 2.95) (Figure 3). A carryover effect was observed in the blank hoof matrix sample at 292.1 > 141.0 transition. It was deemed acceptable, because the residual signal corresponded to a 0.05% carryover for the analyte (and 0.02% for the IS).

These results confirmed the specificity of this method for the identification of TBF and TBF‐d3 in the hoof matrix.

#### 3.2.2 ME

Ion suppression was observed with the analyte and the IS, with a ME of 91.9% ± 4.6% and an IS‐normalised matrix factor of 93.9% ± 2.5% (Table 2).

**TABLE 2:** Matrix effect and IS‐normalised matrix effect of quality controls.

Concentration (ng/mL)	Matrix effect (%, *n* = 3)	IS‐normalised matrix effect (%, *n* = 3)
25	87.7	93.4
75	90.5	92.5
150	97.4	95.8

#### 3.2.3 Extraction Procedure

The average analyte recovery from the porcine hoof matrix varied between 83.5% ± 4.6% and 85.7% ± 7.6% at the different concentrations. Table 3 presents the recovered concentrations and extraction efficiency of the analyte.

**TABLE 3:** Recovered amounts and TBF extraction efficiency.

Spiked amount (μg/mL)	Theoretical concentration after dilution (ng/mL)	Extraction recovery (%)
1	100	85.7 ± 7.6
10	100	83.5 ± 4.4
100	100	83.5 ± 4.6

#### 3.2.4 Calibration Curve Range and Response Linearity

Method linearity was tested over a concentration range from 1 to 200 ng/mL of analyte in ACN:H_2_O (1:1) solvent and in porcine hoof matrix. Both matrices showed a linear response for TBF and good correlation coefficients (*R* ^2^) were obtained for calibration curves (*R* ^2^ > 0.99) (Table 4). An example of the calibration curve is given in the Supporting Information.

**TABLE 4:** Linearity results for both matrices.

Matrix	Linearity range (ng/mL)	Calibration curve	*R* ^2^
ACN:H_2_O (1:1)	1–200	*y* = 1.0128*x* − 0.0041	1.00
Hoof matrix	1–200	*y* = 0.9575 + 0.1944	1.00

#### 3.2.5 LOD and Limit of Quantification

The LLOQ was concluded to be 1 ng/mL by determining the average standard deviation of the difference between the experimental area and the predicted area. The LOD was calculated as 0.3 ng/mL, corresponding to a third of the LLOQ. The method was sensitive enough for the quantification of TBF in the porcine hoof matrix.

#### 3.2.6 Accuracy and Precision

Table 5 shows the results of intra‐day and interday accuracy and precision of the method for the determination of the analyte in the porcine hoof matrix. The mean recovery (accuracy) obtained for the intra‐day measurements ranged between 97.4% and 99.5%, and the precision (RSD) was within the range of 1.2 to 1.9%. For the interday evaluation, the mean recovery was between 95.6% and 99.6%, whereas the RSD ranged from 1.3% to 3.3%. All intra‐day and interday recovery values and RSD values were found to be in an acceptable range.

**TABLE 5:** Accuracy and precision (intra‐day and Interday 1–3) of quality controls in porcine hoof matrix.

[TBF]_theo_ (ng/mL)	Intra‐day [TBF]_meas_ (ng/mL)	RSD (%)	Recovery (%)	Interday 1 [TBF]_meas_ (ng/mL)	RSD (%)	Recovery (%)	Interday 2 [TBF]_meas_ (ng/mL)	RSD (%)	Recovery (%)	Interday 3 [TBF]_meas_ (ng/mL)	RSD (%)	Recovery (%)
25	24.9 ± 0.5	1.9	99.5	24.9 ± 0.6	2.5	99.6	24.8 ± 0.8	3.3	99.3	24.9 ± 0.6	2.2	99.6
75	73.8 ± 0.9	1.2	98.4	74.0 ± 2.4	3.3	98.7	74.1 ± 0.9	1.3	98.8	73.7 ± 1.6	2.2	98.2
150	146.1 ± 2.4	1.6	97.4	143.4 ± 4.6	3.2	95.6	144.3 ± 2.5	1.7	96.2	144.7 ± 4.6	3.2	96.4

### 3.3 Preliminary Study

**FIGURE 4:**
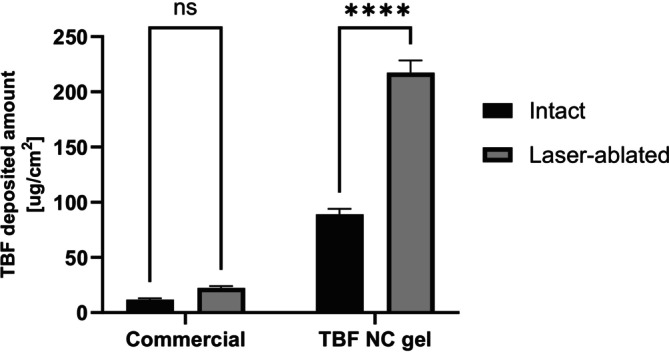
Total deposited amount of TBF following single application of TBF formulations on intact and laser‐ablated porcine hooves (*n* = 3) (****p < 0.0001).

The total quantification of TBF deposited in laser‐ablated and intact porcine hooves following a single application of TBF NC gel and commercial formulations is presented in Figure 4. The amounts of TBF deposited following application of the commercial formulation to intact and laser‐ablated porcine hooves were 11.69 ± 1.15 and 22.30 ± 1.40 μg/cm^2^, respectively. The corresponding values following application of the TBF NC gel were significantly higher (89.04 ± 4.02 and 217.49 ± 8.98 μg/cm^2^, respectively). The RSD values correspond to 10% and 6% for commercial formulation, respectively, 5% and 12% for TBF NC gel. A statistically significant difference between the results obtained with intact and laser‐ablated hooves was only observed for TBF NC gel (*p* < 0.0001).

The results demonstrated that (i) the method could be used to quantify TBF in nail matrix samples, (ii) there was superior TBF deposition in the microporated nail matrix—pointing to the use of fractional laser ablation as a means to enhance ungual TBF bioavailability and hence improve treatment efficacy in vivo (Ortner et al. 2022; Tsai et al. 2016; Vanstone et al. 2017) and (iii) the high drug loading of TBF NC gel enabled significantly greater amounts of TBF to be deposited in the micropores than a conventional (1%) commercially available TBF product.

### 3.4 Novelty of the Method

To date, no detailed validated UHPLC–MS/MS method has been published to quantify TBF in porcine hooves—a useful surrogate for (trans)ungual delivery studies.

Although several liquid chromatographic methods have been published to quantify TBF, these were developed using other biological matrices, including human plasma and human hair (Brignol et al. 2000; de Oliveira et al. 2001; Denouël et al. 1995; Majumdar et al. 2000). As mentioned previously, a single report was found where supplementary data presented details of the method validation in human nails (Davies‐Strickleton et al. 2020). However, the method was designed to quantify multiple compounds under the same settings, rather than specifically optimised for TBF alone. Moreover, the approach involved nail dissolution to obtain lysates for analysis rather than extracting the compounds, and this can increase the amounts of endogenous compounds and interference with the analyte—as mentioned above, harsh conditions can also impact analyte stability.

The present analytical method demonstrated an appropriate sensitivity to quantify concentration ranges between 1 and 200 ng/mL within a runtime of 8 min. In comparison with the aforementioned study, our method presents a similar LLOQ of 1 ng/mL for TBF, but longer runtime (7.2 vs. 2.5 min). Despite that, the straightforward extraction procedure using organic phase enabled a higher recovery of TBF (85.7% ± 7.6% in MeOH:H_2_O [9:1] vs. 38.6 ± 4 in NaOH 5 M), while also improving the analyte structural integrity and minimising the presence of excessive amounts of endogenous compounds compared with lysates. In addition, the drug‐specific method optimisation makes this method suited for future studies focusing on TBF alone.

The preliminary ex vivo study showed the robustness of the validated method to assess TBF deposition following formulation application into laser‐ablated porcine hooves.

## 4 Conclusions and Perspectives

This study presents the first UHPLC–MS/MS method developed and validated to quantify TBF extracted from porcine hooves. The use of porcine hooves as a substitute for human nails provides a cost‐effective and ethical alternative to quantify TBF in an ungual drug delivery ex vivo model, while also offering insights into potential outcomes with onychomycotic toenails, which are significantly different from healthy human nails—notably being considerably thicker. As presented in the preliminary study, the method was used to quantify TBF deposition in intact and laser‐ablated porcine hooves. The objective is to use this analytical method in both preclinical and clinical studies (after suitable fine‐tuning) to study the combination of formulation strategies, such as the use of the TBF NC gel used here, in conjunction with fractional laser ablation to enhance TBF ungual delivery and thereby improve the efficacy of toenail onychomycosis treatments.

## Conflicts of Interest

The authors declare no conflicts of interest.

## Supporting information


**Table S1:** Accuracy and precision (intra‐day and Interday 1–3) of quality controls in ACN:H_2_O (1:1).

## Data Availability

The data that support the findings of this study are available on request from the corresponding author. The data are not publicly available due to privacy or ethical restrictions.
