# Supplementary material for: Bioanalytical UHPLC–MS/MS Method for Quantification of Terbinafine in Ungual Delivery Studies
Source: Biomed Chromatogr. 2026 Feb 12;40(3):e70392. doi: 10.1002/bmc.70392 (PMC12902604; doi:10.1002/bmc.70392)
Supplement: Supplementary file 1 — Table S1: Accuracy and precision (intra‐day and Interday 1–3) of quality controls in ACN:H2O (1:1). [file BMC-40-e70392-s001.docx]

# **Supplementary Data**

**Bioanalytical UHPLC-MS/MS method for quantification of terbinafine in ungual delivery studies**

Benjamin Rossier^1,2^, Emmanuel Varesio^1,2^, Eric Allémann^1,2^, and Yogeshvar N. Kalia^1,2*^

^1^School of Pharmaceutical Sciences, University of Geneva, Rue Michel-Servet 1, 1211 Geneva 4, Switzerland

^2^Institute of Pharmaceutical Sciences of Western Switzerland, Rue Michel-Servet 1, 1211 Geneva 4, Switzerland

***Corresponding author**:

Prof. Yogeshvar N. Kalia,

School of Pharmaceutical Sciences, University of Geneva,

CMU - 1 rue Michel-Servet, 1211, Geneva 4, Switzerland.

E-mail: [yogi.kalia@unige.ch](mailto:yogi.kalia@unige.ch)

## 6.1. Determination of the isotopic contribution of TBF-d3


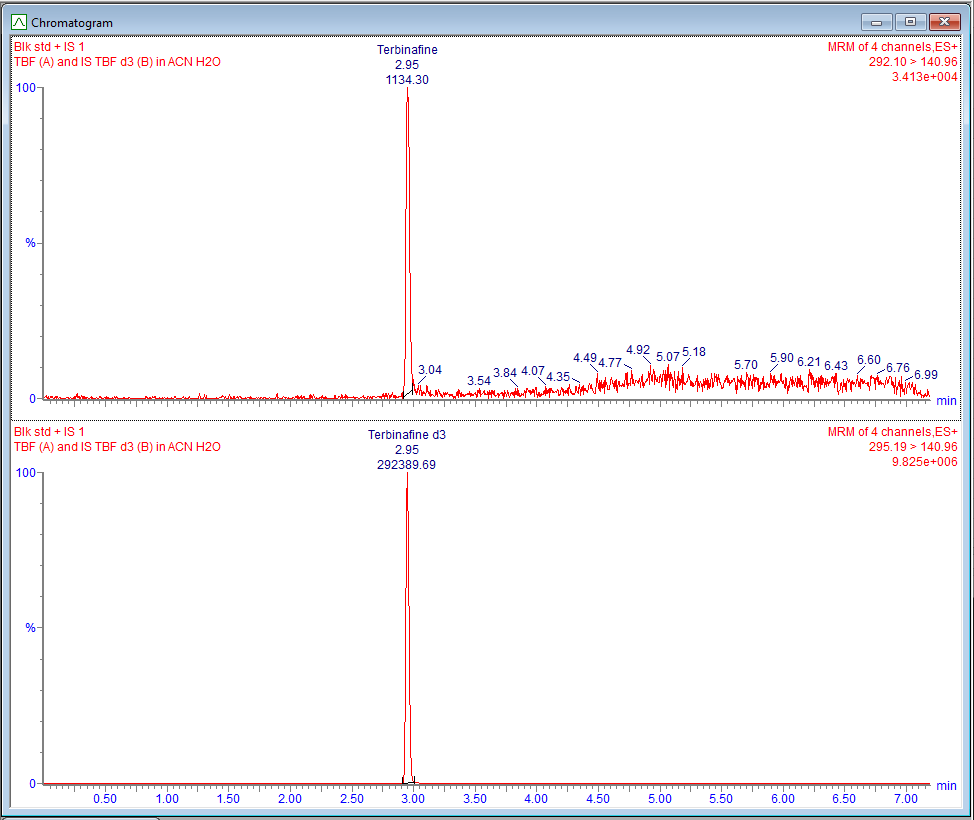


Area-based isotopic contribution of the IS to the TBF signal = (1134.30 / 292389.69) = 0.39%

## 6.2. Example of calibration curve


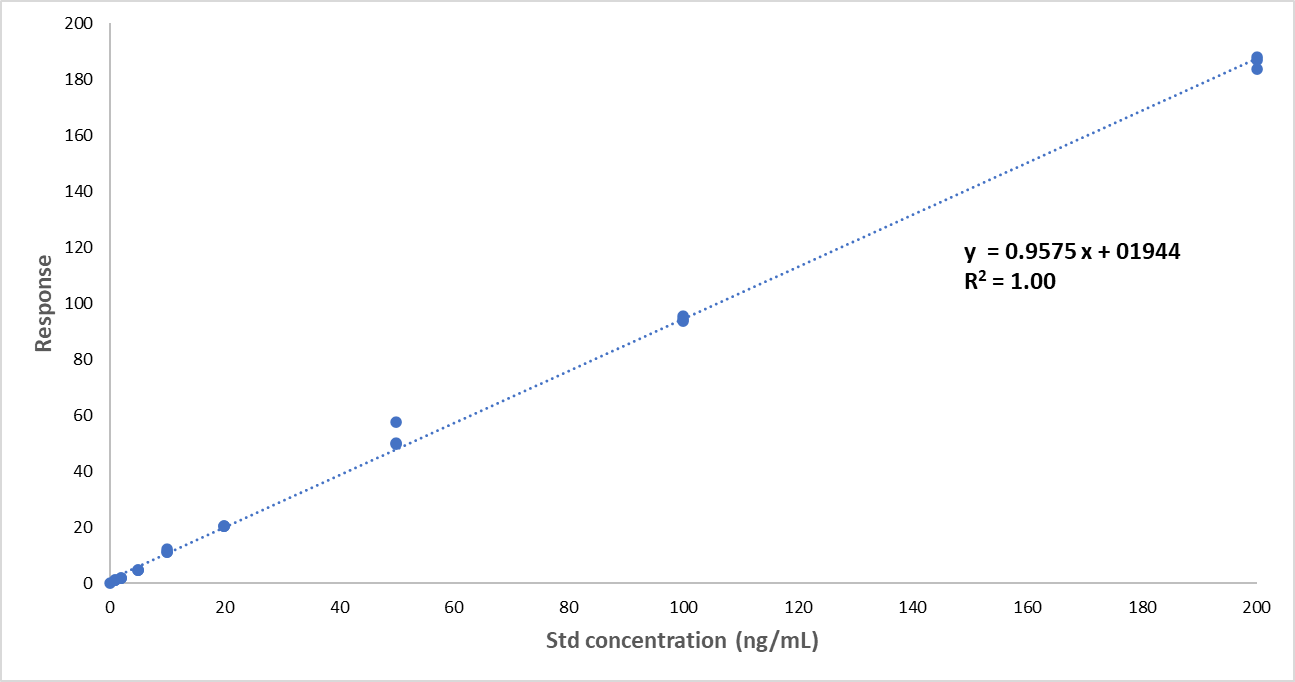


## 6.3. Accuracy and precision of quality controls prepared in ACN:H_2_O (1:1)

**Table S1**. Accuracy and precision (intra-day and inter-day 1, 2 and 3) of quality controls in ACN:H_2_O (1:1)

|  | **Intra-day** |  |  | **Inter-day 1** |  |  | **Inter-day 2** |  |  | **Inter-day 3** |  |  |
| --- | --- | --- | --- | --- | --- | --- | --- | --- | --- | --- | --- | --- |
| **Theor. conc. (ng/mL)** | **Measured conc. (ng/mL)** | **RSD (%)** | **Recovery (%)** | **Measured conc. (ng/mL)** | **RSD (%)** | **Recovery (%)** | **Measured conc. (ng/mL)** | **RSD (%)** | **Recovery (%)** | **Measured conc. (ng/mL)** | **RSD (%)** | **Recovery (%)** |
| 25 | 25.4 ± 0.3 | 1.2 | 101.7 | 25.3 ± 0.4 | 1.6 | 101.1 | 25.6 ± 0.2 | 0.9 | 102.3 | 25.6 ± 1.0 | 3.9 | 102.3 |
| 75 | 75.7 ± 1.7 | 2.3 | 100.9 | 75.0 ± 1.7 | 2.2 | 100.0 | 74.9 ± 3.0 | 4.0 | 99.9 | 73.4 ± 1.0 | 1.4 | 97.9 |
| 150 | 144.4 ± 1.4 | 1.0 | 96.2 | 144.2 ± 3.6 | 2.5 | 96.1 | 146.8 ± 2.1 | 1.4 | 97.9 | 147.4 ± 2.8 | 1.9 | 98.2 |
